# Effects of etidronate on the *Enpp1^−/−^* mouse model of generalized arterial calcification of infancy

**DOI:** 10.3892/ijmm.2015.2212

**Published:** 2015-05-15

**Authors:** CARMEN HUESA, KATHERINE A STAINES, JOSE LUIS MILLÁN, VICKY E MacRAE

**Affiliations:** 1 Roslin Institute and R(D)SVS, The University of Edinburgh, Edinburgh, UK; 2 Sanford Children’s Health Research Center, Sanford-Burnham Medical Research Institute, La Jolla, CA, USA

**Keywords:** mineralization, vascular calcification, ectonucleotide pyrophosphatase/phosphodiesterase 1, etidronate, generalized arterial calcification of infancy

## Abstract

Generalized arterial calcification of infancy (GACI) is an autosomal recessive disorder of spontaneous infantile arterial and periarticular calcification which is attributed to mutations in the ectonucleotide pyrophosphatase/phosphodiesterase 1 (*Enpp1*) gene. Whilst the bisphosphonate, etidronate, is currently used off-label for the treatment for GACI, recent studies have highlighted its detrimental effects on bone mineralisation. In the present study, we used the *Enpp1^−/−^* mouse model of GACI to examine the effects of etidronate treatment (100 *µ*g/kg), on vascular and skeletal calcification. Micro-computed tomography (*µ*CT) analysis revealed a significant decrease in trabecular bone mass, as reflected by the decrease in trabecular bone volume/tissue volume (BV/TV; %), trabecular thickness, trabecular separation, trabecular number and pattern factor (P<0.05) in the *Enpp1^−/−^* mice in comparison to the wild-type (WT) mice. Mechanical testing revealed that in the WT mice, treatment with etidronate significantly improved work to fracture and increased work post-failure (P<0.05, in comparison to the vehicle-treated WT mice). This significant increase, however, was not observed in the *Enpp1^−/−^* mice. Treatment with etidronate had no effect on bone parameters in the WT mice; however, the *Enpp1^−/−^* mice displayed an increased structural model index (SMI; P<0.05). We used a recently developed 3D *µ*CT protocol to reconstruct and quantify the extensive aortic calcification in *Enpp1^−/−^* mice in comparison to the WT mice. However, treatment with etidronate did not prevent *de novo* calcification, and did not arrest the progression of established calcification of the aorta.

## Introduction

Generalized arterial calcification of infancy (GACI) is an autosomal recessive disorder of spontaneous infantile arterial and periarticular calcification (1–3). This life-threatening disease is caused by loss-of-function mutations in the ectonucleotide pyrophosphatase/phosphodiesterase 1 (*ENPP1*) gene, a key regulator of biomineralisation and vascular calcification (4–8).

ENPP1 is a cell-surface glycoprotein enzyme that functions in synergy with the anklyosis protein (ANK) to respectively form and intracellularly channel inorganic pyrophosphate (PP_i_), an inhibitor of hydroxyapatite formation, from nucleoside triphosphates (9–12). The extracellular concentration of PP_i_ is further influenced by tissue non-specific alkaline phosphatase (TNSALP), another cell-surface enzyme located on the cell membrane of osteoblasts and chondrocytes, as well as on the membranes of their matrix vesicles (MVs) (13). TNSALP exerts its effects by hydrolysing PP_i_ reducing the concentration of this mineralisation inhibitor and establishing a phosphate (Pi)/PP_i_ ratio permissive for the formation of hydroxyapatite crystals (14–17). Phosphatase, orphan 1 (PHOSPHO1) is another essential phosphatase, located within osteoblast- and chondrocyte-derived MVs with high phosphohydrolase activity toward phosphoethanolamine and phosphocholine (18–22), which contributes Pi for the initiation of skeletal mineralisation. Together, ENPP1, ANK, TNSALP and PHOSPHO1 control the Pi/PPi ratio conducive to physiological skeletal mineralisation. Thus, ENPP1 in GACI reduces extracellular PP_i_ levels and predisposes to ectopic calcification. This was further exemplified in a previous study of ours, in which we determined that vascular smooth muscle cells from mice deficient in *Enpp1* have increased TNSALP levels (23).

In naturally occurring mouse models, the link between defective *Enpp1* expression and altered mineralisation was initially demonstrated in ‘tiptoe walking’ (*ttw/ttw*) mice (24–28). These animals are homozygous for a G→T substitution resulting in the introduction of a stop codon in the NPP1 coding sequence. The subsequent truncated protein leads to the loss of a vital calcium binding domain and two putative glycosylation sites (25). The phenotype of this mouse includes the postnatal development of progressive ankylosing intervertebral and peripheral joint hyperostosis, as well as spontaneous arterial and articular cartilage calcification and increased vertebral cortical bone formation (24–28). Transgenic mice that are homozygous for a disruption in exon 9 of the *Enpp1* gene (*Enpp1^−/−^ mice*) exhibit abnormalities that are almost identical to those present in *ttw/ttw* mice (29). These include decreased levels of extracellular PP_i_, with phenotypic characteristics, including significant alterations in bone mineralisation in long bones and calvariae, and pathological, severe peri-spinal soft tissue and arterial calcification (30–32).

Effective treatment for infants and young children with GACI is critical as without it, 85% of patients succumb to the disease within 6 months of age. First used off-label in the treatment of *Fibrodysplasia ossificans progressiva*, the ‘first generation’ bisphosphonate, etidronate (EHDP; ethane-1-hydroxy-1,1-diphosphonic acid, also known as 1-hydroxyethylidene-bisphosphonate) is an analogue of PP_i_ and has also been used in the treatment of GACI. Bisphosphonates are potent inhibitors of osteoclast activity, and are widely used in clinical practice to prevent the bone loss associated with conditions, such as Paget’s disease, metastatic bone disease and osteoporosis (33). The inhibitory effects of bisphosphonates on osteoblast function have also been demonstrated (34–37).

In 2008, a retrospective observational analysis of 55 patients with GACI revealed survival beyond infancy with etidronate therapy (38) corroborated by a recent study highlighting that 15 out of 22 GACI survivors received etidronate (39). However, studies on uremic rats have suggested that the administration of etidronate may not be able to prevent arterial calcification without inhibiting bone formation (40). Furthermore, a recent case report has highlighted the profound inhibition of skeletal mineralisation with paradoxical joint calcifications following protracted etidronate therapy in a 7-year-old boy with GACI (41). Taken together, these findings have led us to herein assess the effects of etidronate on bone architecture and arterial calcification in the *Enpp1^−/−^* mouse model of GACI.

## Materials and methods

*Animals. Enpp1*^−/−^ and wild-type (WT) mice were generated and maintained as previously described (1,5,29,42). Male mice were administered etidronate at 100 *µ*g/kg, intraperitoneally twice a week from 11 to 22 weeks of age. The dosage of etidronate used in this study was based on the dose reported in a previously study (43). Animals were administered saline as a placebo (vehicle treatment). All animals were weighed once a week. The animals were sacrificed at 22 weeks of age and the tissues were dissected for further analysis. All animal experiments were approved by The Roslin Institute’s Animal Users Committee and the animals were maintained in accordance with the UK Home Office guidelines for the care and use of laboratory animals (PIL number DD 60/3828).

### Preparation of tissue

The aortae and tibiae were dissected as previously described (5). The aortae were fixed in 10% neutral buffered formalin (NBF) for 48 h before being transferred to 70% ethanol. The tibiae were immediately frozen in distilled water pending analysis.

### Micro-computed tomography (µCT) of the aortae

Prior to scanning, the aortae were immersed for a minimum period of time (10 min) in a macro-molecular iopamidol-based contrast agent (Niopam 300; Brako UK Ltd., High Wycombe, Buckinghamshire, UK) diluted 1:4 in water as previously described (44). To allow tissue differentiation, aortic luminae were filled with corn oil and the aortae were submersed in oil for the duration of the scan. Tissues were imaged using a Skyscan 1172 X-Ray Microtomograph (Bruker Daltonics, Brussels, Belgium). Sequential high-resolution scans were acquired using a rotation step of 0.3° with the averaging of 3 frames at each step, applying a 0.5-mm aluminium filter, with an X-ray source set at 60 kV and 167 *µ*A, and with an isotropic voxel size of 7 *µ*m. The scans were reconstructed using NRecon (Bruker Daltonics). Noise in the reconstructed images was reduced by applying a median filter (radius = 1). The region of interest was selected to be the aortic arch, 200 lices (1.4 mm) under the subclavian artery. Soft and calcified tissue was identified by thresholding using CTAn software (Bruker Daltonics).

### µCT of the tibiae

High-resolution scans with an isotropic voxel size of 5 *µ*m were acquired with a *µ*CT system (60 kV, 0.5 mm aluminium filter, 0.6° rotation, Skyscan 1172; Bruker Daltonics). Scans were reconstructed using NRecon software (Bruker Daltonics). A 1,000-*µ*m section of the metaphysis 250 *µ*m off the reference plate was taken for analysis of the trabecular bone. The base of the growth plate was used as a standard reference point. A 250-*µ*m metaphysis section of the mid-diaphysis, 1,500 *µ*m below the reference plate, was scanned for the analysis of cortical structure. Data were analysed with CTAn software (Bruker Daltonics). The following parameters were analysed using CTAn software (Bruker Daltonics): percentage bone volume/trabecular bone volume (%BV/TV), trabecular number (Tb.N;/mm), trabecular patten factor (Tb.Pf), bone mineral density (BMD; g/cm^3^), trabecular thickness (Tb.Th; mm), trabecular separation (Tb. Sp) and the structure model index (SMI) were evaluated. In the cortical bone, %BV/TV, BMD (g/cm^3^), cortical thickness, cross-sectional area (mm^2^), the percentage of closed pores and polar moment of inertia (mm^4^) were evaluated.

### Mechanical testing

Mechanical testing of the cortical bone was carried out using a Zwick materials testing machine (Zwick Armaturen GmbH, Ennepetal, Germany) and data were analysed as previously described (45). The span was fixed at 6.0 mm. The cross-head was lowered at 1 mm/min and data were recorded after every 0.1 mm change in deflection. Each bone was tested to fracture. Failure and fracture points were identified from the load-extension curve as the point of maximum load and where the load rapidly decreased to zero, respectively. The maximum stiffness was defined as the maximum gradient of the rising portion of this curve, and the yield point, the point at which the gradient reduced to 95% of this value. Both values were calculated from a polynomial curve fitted to the rising region of the load-extension curve.

### Serum marker analysis

To determine differences in bone formation and resorption, plasma serum was collected from the mice at 22 weeks of age. A sandwich ELISA P1NP (IDS Ltd., Boldons, UK) and a C-terminal telopoptide of type I collagen (CTx) ELISA kit (RatLaps™; IDS) were used respectively, and analyses were performed according to the manufacturer’s instructions.

### Statistical analysis

General linear model analysis, the Student’s t-test, the Mann-Whitney non-parametric test and Pearson’s correlation anlaysis were used to assess the data where appropriate. All data are expressed as the means ± SEM. Statistical analysis was performed using SPSS (IBM Software, New York, NY, USA). A value of P<0.05 was considered to indicate a statistically significant difference.

## Results

### Enpp1^−/−^ mouse growth phenotype

**Figure 1:**
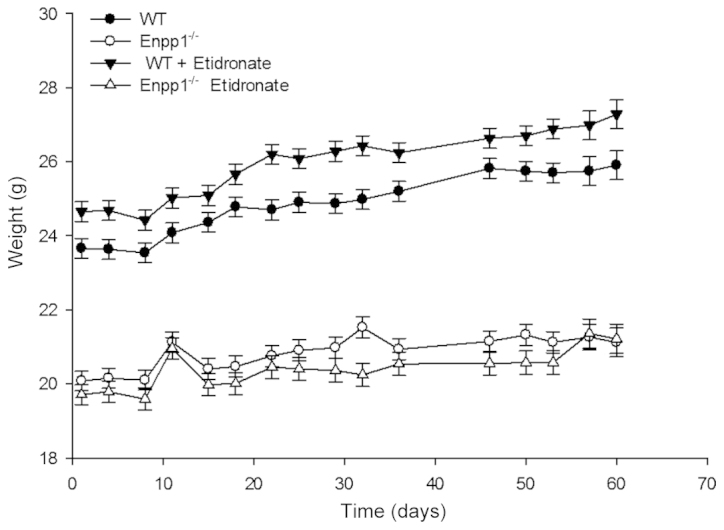
Body weight of 11-week-old male *Enpp1^−/−^* and wild-type (WT) mice taken for 60 days from the day of administration of etidronate (day 0). Data are expressed as the means ± SEM.

In initial experiments, we examined whether the treatment of *Enpp1^−/−^* and WT mice with 100 *µ*g/kg etidronate affects their growth. In accordance with our previous study, the *Enpp1^−/−^* mice exhibited a reduced growth in comparison to the WT mice (18.4% smaller than the age-matched WT controls; P<0.05) (5). Intraperitoneal injections of etidronate had no effect on the total body weight of the WT mice, nor the *Enpp1^−/−^* mice in comparison to the respective vehicle-treated mice (Fig. 1). Notably, the *Enpp1^−/−^* mice appeared to lose weight from approximately 12 weeks of age, which may be a consequence of their limited movement due to excessive joint calcification (Fig. 1) (1,5).

### Aortic calcification

**Figure 2:**
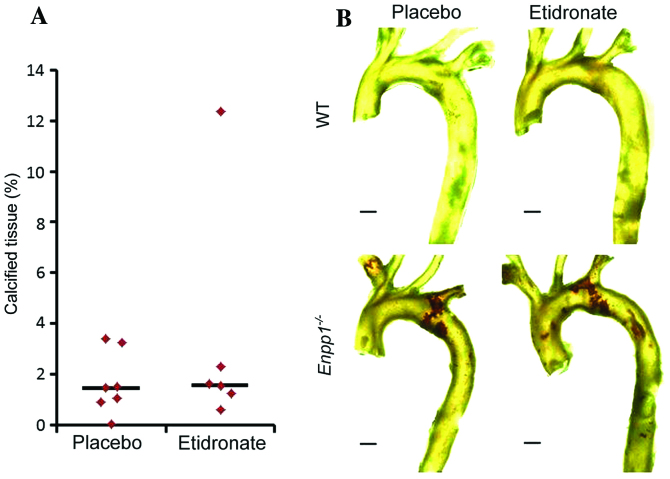
(A) Quantification (% of calcification) of calcium deposition in the aortae of 22-week-old *Enpp1^−/−^* mice. A standardised region of calcium deposition (400 slices from the subclavian artery) was selected and revealed no significant differences between the placebo (vehicle; saline)- and etidronate-treated groups. (B) Three-dimensional volumetric reconstructions of aortae from 22-week-old wild-type (WT) placebo-treated, WT etidronate-treated, *Enpp1^−/−^* placebo-treated, and *Enpp1^−/−^* etidronate-treated mice. Calcification is indicated by brown colouring. Bar represents 0.5 mm.

We have previously demonstrated that *Enpp1^−/−^* mice exhibit arterial calcification from 11 weeks of age (31). In this study, we employed our recently developed three-dimensional (3D) *µ*CT protocol (44) for the quantification of aortic calcification to examine the effects of treatment with etidronate on mice lacking *Enpp1*. As expected, the *Enpp1^−/−^* mice exhibited extensive aortic calcification in comparison to the WT mice at 22 weeks of age (Fig. 2B). However, treatment with etidronate did not prevent *de novo* calcification, and did not arrest the progression of established calcification of the aorta in these mice (Fig. 2).

### µCT analysis of bone microarchitecture

*Enpp1^−/−^* mice have previously been reported to display reduced mineral content in bone, with a reduction in bone volume fraction and trabecular thickness (5). The present study extended these observations by fully examining the effects of the administration of etidronate on the bone phenotype of *Enpp1^−/−^* mice. *µ*CT analysis of the tibiae from *Enpp1^−/−^* mice in comparison to those from WT mice (both vehicle-treated) at 22 weeks of age revealed a significant decrease in trabecular bone mass, as reflected by a decrease in %BV/TV, trabecular thickness and trabecular number (P<0.05; Table I). Moreover, we observed a significant decrease in cortical parameters in the tibiae of the 22-week-old *Enpp1^−/−^* mice in comparison to the age-matched WT mice, except for cortical porosity (P<0.05; Table II). Treatment with etidronate had no significant effect on cortical or trabecular bone parameters in the WT mice (Tables I and II). In the *Enpp1^−/−^* mice, treatment with etidronate resulted in an increase in trabecular number and %BV/TV, as reflected by the significant decrease in trabecular separation (P<0.05, in comparison to the vehicle-treated *Enpp1^−/−^* mice) (Table I). The *Enpp1^−/−^* mice treated with etidronate did show a significant decrease in SMI [quantification of the plate- or rod-like geometry of trabecular structures, as previously described (46)] compared to the vehicle-treated *Enpp1^−/−^* mice (P<0.05; Table I).

### Mechanical testing

The changes in bone geometry observed as a result of treatment with etidronate in the *Enpp1^−/−^* mice are likely to alter the biomechanical properties of long bones. In order to examine this hypothesis, we carried out 3-point bending analysis of the tibiae. Mechanical testing revealed a significant decrease in all mechanical parameters examined (stiffness, load at failure, work to failure, load at fracture, work to fracture, and yield) except work post-failure, in the *Enpp1^−/−^* mice compared to the WT mice at 22 weeks of age (Table III; P<0.05), reflecting reduced bone strength and stiffness as we have previously reported (5). In the WT mice, treatment with 100 *µ*g/kg etidronate significantly improved work to fracture and increased work post-failure (Table III; P<0.05, in comparison to the vehicle-treated WT mice); this suggests that more energy is required to fracture these etidronate-treated bones in comparison to the vehicle-treated bones. This significant increase, however, was not observed in the *Enpp1^−/−^*mice treated with etidronate (Table III).

### Plasma biochemical markers

**Figure 3:**
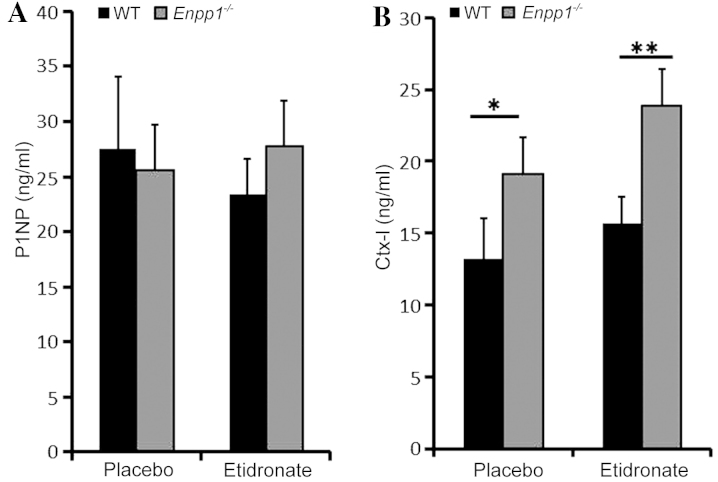
Serum marker analysis in *Enpp1^−/−^* and wild-type (WT) mice. (A) P1NP, a marker of bone formation; (B) CTx (RatLaps™), a marker of bone resorption. Results are expressed as the means ± SEM. ^*^P<0.05; ^**^P<0.01.

The level of osteoblast and osteoclast activity was assessed by ELISA of serum taken from the etidronate-treated and vehicle-treated 22-week-old male *Enpp1^−/−^* and WT mice. The plasma concentrations of P1NP, a marker of bone formation, were unaltered in the *Enpp1^−/−^* and WT mice at 22 weeks of age (Fig. 3A). Moreover, no significant differences in bone formation were observed upon the administration of etidronate in the WT or *Enpp1^−/−^* mice (Fig. 3A). The plasma concentrations of CTx, a marker of bone resorption, were increased in the *Enpp1^−/−^* mice in comparison to the WT mice in both the etidronate- and vehicle-treated mice (P<0.05; Fig. 3B). This is in concordance with our previous observation of this marker in *Enpp1^−/−^* mice (5). However, there were no significant differences observed between the etidronate- and vehicle-treated mice in either parameter (Fig. 3).

## Discussion

Studies have associated treatment with bisphosphonates, chiefly etidronate, with improved survival in patients with GACI, an autosomal recessive disorder of spontaneous infantile arterial and periarticular calcification which is attributed to mutations in the *ENPP1* gene (38). Animal models have proven to be key to the understanding of pathological ectopic mineralisation (4,47). In particular, the *Enpp1^−/−^* mouse is of particular importance in advancing our understanding of GACI. Thus, the present study was undertaken to determine the effects of etidronate on *Enpp1^−/−^* mice.

Our data confirm and extend those of our previous study (5), demonstrating that tibiae from *Enpp1^−/−^* mice have a reduced trabecular bone mass and cortical thickness in comparison to WT mice, which explains the altered bone mechanical properties noted in the present study. This, therefore, is consistent with the depletion of NPP1 activity reducing extracellular PP_i_ to abnormally low levels, resulting in insufficient PP_i_ a substrate for TNAP to generate P_i_ for normal mineral formation. In the present study, we also used our novel 3D *µ*CT protocol to provide evidence of the severe hypermineralisation of the arteries in *Enpp1^−/−^* mice, consistent with reduced extracellular PP_i_ levels predisposing the vascular system to ectopic calcification.

Bisphosphonates are typically prescribed for the treatment of osteoporosis and to reduce fracture risk, preventing bone loss primarily by the inhibition of osteoclast function (33). However, there is evidence to suggest that bisphosphonates impair the anabolic response of bone to parathyroid hormone (48), inhibiting osteoblast function and suppressing bone formation (34–37). Furthermore, the hydrolysis-resistant P-C-P motif of bisphosphonates resembles the TNSALP susceptible core of PP_i_, (49) permitting bisphosphonates to impair calcium phosphate crystallisation (50). First used off-label in the treatment of *Fibrodysplasia ossificans progressiva*, the ‘first generation’ bisphosphonate, etidronate, is an analogue of PP_i_, and whilst it is currently being investigated as a treatment for GACI, a number of studies have highlighted a number of detrimental effects with this approach, including the development of rickets or osteomalacia during the protracted administration of etidro-nate (40,41,51–56).

In this study, to clarify the effects of bisphosphonates on GACI, we treated WT and *Enpp1^−/−^* mice with 100 *µ*g/kg etidro-nate. In the WT mice, treatment with etidronate had no effect on cortical or trabecular parameters as determined by *µ*CT analysis. Despite this, treatment with etidronate significantly improved work to fracture and increased work post-failure, thus suggesting that more energy is required to fracture these bones in comparison to the vehicle-treated bones. The assessment of bone architecture in *Enpp1^−/−^* mice treated with etidronate revealed a significant decrease in SMI, a method for the determination of the plate- or rod-like geometry of trabecular structures. This change in bone geometry did not, however, affect the bone mechanical properties. Consistent with this, the assessment of plasma markers of bone formation and resorption revealed that this dosage of etidronate did not significantly affect the bone remodelling process in the *Enpp1^−/−^* mice, nor in WT mice. These findings add further support to those of a recent *in vivo* study using mice, which also reported no effects of the administration of etidronate on bone resorption (57). Furthermore, the anti-resorptive effects of alendronate, risedro-nate and minodronate were revealed to be 1,000-, 3,300- and 10,000-fold greater than those of etidronate, respectively (57).

Surprisingly, and in contrast to previous data on rats with experimental renal failure (40), treatment with etidronate did not prevent *de novo* calcification, and did not arrest the progression of established calcification of the aorta in our *Enpp1^−/−^* mice. Taken together, these data suggest that the skeleton here is displaying an expected response to etidronate treatment, but this is not yet toxic to aortic calcification.

The mild effects of etidronate observed in this study may be explained by the dosage used, the frequency of administration and/or species differences. Future studies may aim to investigate a higher dosage and/or daily administration of etidronate, as previously it has been shown that high doses of etidronate significantly reduce mineralisation (58). Furthermore, in the present study, treatment of the mice commenced when the mice were 11 weeks of age, the point at which calcification is observed in this model (5). However, this initiation of treatment may have been too late to resolve underlying, pre-existing calcifications and additional investigation into this would allow further conclusions to be drawn.

In conclusion, despite the changes in bone microarchitecture, we did not observe an inhibition of aortic calcification or bone formation in the *Enpp1^−/−^* mice treated with etidronate. Additional studies are, therefore, required to fully determine whether etidronate is the most appropriate therapy for the treatment of GACI.

## Figures and Tables

**Table I:** *µ*CT analysis of trabecular bone in male placebo (vehicle)- and etidronate-treated *Enpp1^−/−^* and WT mice.

	WT		*Enpp1^−/−^*	
	Placebo	Etidronate	Placebo	Etidronate
BV/TV (%)	8.79±1.59	8.75±1.79	3.39±1.06^a^,^e^	4.63±1.57^a^,^e^
BMD (g/cm^3^)	0.139±0.021	0.13±0.024	0.042±0.020^a^,^e^	0.062±0.025^a^,^e^
Tb.Th (*µ*m)	60.62±4.31	57.34±8.34	48.35±3.08^a^,^e^	47.99±3.54^a^,^d^
Tb.Sp (*µ*m)	298.24±12.68	290.47±26.56	363.66±48.07^a^,^d^	319.66±48.44^b^,^c^
Tb.N	0.00146±0.00023	0.0015±0.00029	0.00070±0.00024^a^,^e^	0.00097±0.00034^a^,^e^
Tb.Pf	0.027±0.0026	0.026±0.0033	0.042±0.0036^a^,^e^	0.0392±0.0053^a^,^e^
SMI	2.48±0.11	2.33±0.23	2.78±0.093^a^,^d^	2.69±0.22^a^,^e^
DA	2.30±0.19	2.08±0.15	2.42±0.28	2.39±0.31^a^,^c^

Results are expressed as the means ± SEM.

a Significant difference compared to wild-type (WT) mice;

b significant difference compared to placebo-treated mice.

c P<0.05;

d P<0.01;

e P<0.001. *µ*CT, micro-computed tomography; BV/TV, bone volume/trabecular bone volume; BMD, bone mineral density; Tb.Th, trabecular thickness; Tb.Sp, trabecular separation; Tb.N, trabecular number; Tb.Pf, trabecular patten factor; SMI, structure model index; DA, degree of anisotropy.

**Table II:** *µ*CT analysis of cortical bone in male placebo (vehicle)- and etidronate-treated *Enpp1^−/−^* and WT mice.

	WT		*Enpp1^−/−^*	
	Placebo	Etidronate	Placebo	Etidronate
Co.BMD (g/cm^3^)	1.18±0.018	1.18±0.020	1.20±0.024	1.20±0.011^a^
Co.Po (%)	53.27±7.26	54.79±5.64	61.75±3.71^a^,^c^	63.11±4.85^a^,^c^
Co.Th (*µ*m)	197.90±1.91	195.64±3.84	175.98±5.96^a^,^d^	169.86±15.92^a^,^d^
Co.Area (*µ*m^2^)	5.14E+06±5.57E+05	5.09E+06±3.72E+05	4.47E+06±4.2E+05^a^,^e^	4.17E+06±4.3E+05^a^,^e^

Results are expressed as the means ± SEM.

a Significant difference compared to wild-type (WT) mice;

b significant difference compared to placebo-treated mice.

c P<0.05;

d P<0.01;

e P<0.001. *µ*CT, micro-computed tomography; cortical bone mineral density; Co.BMD, cortical bone mineral density; Co.Po; cortical porosity; Co.Th, cortical thickness; Co.Area, cortical area.

**Table III:** Measurements of tibia mechanical properties in male placebo (vehicle)- and etidronate-treated *Enpp1^−/−^* and WT mice.

	WT		*Enpp1^−/−^*	
	Placebo	Etidronate	Placebo	Etidronate
Stiffness (N/mm)	44.56±7.08	48.82±23.43	22.42±7.19^a^,^d^	22.52±6.64^a^,^e^
Load at failure (N)	17.22±2.90	15.34±4.13	9.21±1.38^a^,^e^	8.89±1.84^a^,^e^
Work to failure (J)	0.0066±0.00066	0.0064±0.0019	0.0033±0.00081^a^,^e^	0.0035±0.00092^a^,^e^
Load at fracture (N)	1.72±0.29	1.53±0.41	0.92±0.14^a^,^e^	0.89±0.18^a^,^e^
Work to fracture (J)	0.0069±0.00087	0.0086±0.0019^b^,^c^	0.0038±0.0011^a^,^e^	0.0044±0.00082^a^,^e^
Work post-failure (J)	0.00037±0.00041	0.0023±0.0014^b^,^d^	0.00055±0.00082	0.00088±0.0012^a^,^c^
Yield (N)	14.41±3.95	12.97±4.42	6.96±1.45^a^,^e^	7.11±1.79^a^,^d^

Results are expressed as the means ± SEM.

a Significant difference compared to wild-type (WT) mice

b significant difference compared to placebo-treated mice;

c P<0.05;

d P<0.01;

e P<0.001.
